# End-of-life decisions and ethics on the big screen: reflecting narratives of ‘a life fully lived’

**DOI:** 10.1007/s11019-025-10296-1

**Published:** 2025-09-26

**Authors:** Franziska Wagensonner, Antonia Sahm, Andreas Frewer

**Affiliations:** Professorship for Medical Ethics, Friedrich-Alexander-Universität Erlangen-Nürnberg, 91054 Erlangen, Germany

**Keywords:** Bioethics, End-of-life, Film, Assisted dying, Euthanasia, Age

## Abstract

The question of what constitutes a good life, whether a human existence is considered fulfilling and how to respond to a life perceived as no longer worth living has long been one of the great inquiries of medical ethics. With the increasing liberalization of various forms of assisted dying worldwide, these fundamental questions are gaining renewed relevance. An emerging field of interest explores films as sociocultural laboratories, offering an intriguing approach to a more nuanced perspective on personal narratives. Applied to the subject of end-of-life decisions this practice turns abstract constructs such as the quest for a meaningful life into tangible plotlines and vivid case studies. Far more than conceptual discussions about morally right or wrong, the storyline on screen enables the viewer to gain a deep and unique insight into the personal life and contextual embeddedness of protagonists struggling with end-of-life decisions. This paper aims to explore the idea and narrative of ‘a life fully lived’ in the movies focussing on end-of-life decisions. It focuses on the implications, demands, and influences on choices concerning death and dying using the example of ten of the most impactful and most debated movies featuring end-of-life decisions. Using film analysis, commonly held assumptions and value judgments that influence public discourse about end-of-life decisions are to be revealed and made accessible for ethical reflection.

## A cinematic approach to the ethics of end-of-life decisions

*Why do we have to go through all this, like we’re criminals?*

*Shouldn’t dying people have the right to end their lives with dignity?*

*They will, once our teetering health system completely collapses.* 1

This quote comes from the 2024 Spanish drama *The Room Next Door*, starring Tilda Swinton as Martha, a former war correspondent suffering from terminal cancer, and Juliane Moore as her long-lost friend Ingrid. Ingrid agrees to stay with Martha ‘in the room next door’ in a secluded country home during her final days as she prepares for her planned suicide. The quote is taken from a conversation between Ingrid and their mutual friend Damian, who, upon learning of the situation, advises Ingrid concerning legal affairs to help obscure her involvement in Martha’s death. As they discuss the complex intersections of politics surrounding assisted suicide and euthanasia, the impact of climate change on the healthcare system and the demographic shifts, Martha takes her own life.

Although many viewers might not watch the headline-making new movie *The Room Next Door* by Spanish cinema luminary Pedro Almodóvar^2^ or go to the movies with the specific aim to gain ethical knowledge about end-of-life decisions, we all do learn something about death, dying and our own fears or values while watching someone struggling with these decisions on screen. There is an abundance of famous movies concerning difficult biomedical issues and there are few movies as heatedly debated in the scientistic community as those featuring ethically difficult questions about the end of life. An important outlook on the increasingly significant role taken by films in our cultural identity in general is their capacity to both reflect and amplify contemporary societal developments or warn of problematic tendencies, which comes with a heightened awareness for its use in medical ethics (Drukarczyk et al. 2014; Lesch 2022).^3^ Far more than conceptual discussions about morally right or wrong decisions, the storyline on screen enables the viewer to gain a deep and unique insight into the personal life and emotional relations of protagonists struggling with common bioethical issues. This practice turns abstract constructs such as an individual’s autonomy over their own death into tangible plotlines that in the best case activate viewers’ empathy. That way it capacitates them to approach the topic intuitively and measure the film’s interpretation against their own ideas of morality. Empathy and empathic identification with a dying character therefore enables viewers to confront the inevitability of their own death, which – so to speak – nobody can experience and live to reflect upon (Schmidt and Roser 2015). In the worst case, however, there is also a significant danger of creating or reinforcing false assumptions about multi-layered subjects like palliative care, end-of-life decisions and the legal and/or healthcare system, as these topics are often alien to the everyday life of the general public. The inevitable reduction of complex ethical dilemmas due to dramaturgic effect as well as the technical limitation of a 90-minute feature film contain the potential to foster a unilateral discussion of the topic (Rubenfeld and Sulmasy 2022), thus influencing the public view towards a specific subject in an unbalanced way, creating unrealistic expectations or even fear. Through identification with the character the direct comparison with our own circumstances of life might also create suggestive pressure, for example, towards accepting assisted dying in old age or in the event of incurable illness or disability. In either case, the audience partakes in a vivid experiment of what complex ethical dilemmas look like in specific cases that allows them to experience how emotions, personal relations, circumstances, and values can shape expectations and influence decisions.

Television itself is a medium of relevance to adults’ daily routines, serving as key source of both information and entertainment. A survey by Nielsen National TV Panel found that people over the age of 18 in the United States spend an approximate average of 4.9 h watching TV daily, taking up a significant amount of time and thus featuring prominently among their pastimes (Fingerman et al. 2022). In short, these findings indicate that film and television formats are key sources of (subconscious) information for most of society. Typical feature films can therefore provide us with highly illuminating material to aid reflection on the images of autonomy, age and illness transmitted in our culture and on options for different forms of assisted dying.

Their relevance to the latter topic is particularly acute in view of the limited knowledge among most of the general population in multiple countries, and indeed among significant swathes of the medical and nursing professions, around what assisted dying is, the critical differences in its various forms, and its availability in the real world (Kopp 2009; Gu and Cheng 2016; Anneser et al. 2016). These findings highlight the potential benefit of reflecting on common themes in the storylines of end-of-life decisions at the movies and their potency to raise awareness for problematic tendencies in the non-fictional world. This essay therefore aims to draw attention to common threads in some of the most influential feature films concerning end-of-life decisions. Movies play a significant role in shaping personal and public perceptions and attitudes, including those related to issues of health, death and ethical decision-making (Lesch 2022; Wulff 2005; Shapshay 2009).^4^ Given this well-documented influence, analysing the portrayal of certain circumstances and motifs in films can offer valuable insights into how these depictions shape our understanding of complex issues such as decision-making in end-of-life care. The paper therefore seeks to investigate central narrative elements in the depiction of ethical decision-making related to end-of-life issues, which can subsequently influence our perception of the topic. It should be considered, that especially with famous productions that gain international attention, as is the case with most award-winning movies, the content will be consumed from spectators in different contexts and its perception may differ depending on the predominant cultural norms, current medical ethos and different legislative in various countries. Therefore, the possible impact a movie or rather a stream of movies can have on the public debate can differ starkly depending on the background but also the timely context regarding important steps in the social or legal debate. In our increasingly globalised and connected world however the public debate is not limited by the borders of states or legislative anymore, so that it is helpful to approach the ethical implications with a more comprehensive understanding of society.

By examining how different forms of assisted dying are portrayed, this analysis shows how the concept of ‘a life fully lived’ is central to cinematic representations of end-of-life decisions and explores how certain societal or structural conditions, as portrayed in the movies, can shape – or hinder – the capacity to perceive a life as worth living. To better approach the potential ethical implication of movies’ influence concerning important public discussion around the subject of assisted dying, the main focus of the analysis concerns the content findings of the debated movies, especially concentrating on dialogue and plotline development. It should be acknowledged, that for the overall impression and level of engagement a cinematic production delivers to its viewers other factors such as special effects, lighting and the arrangement of angles can guide the spectators’ emotions and expectations and therefore also deliver a more subtle influence on perception. While occasional sidenotes to notable technical peculiarities are mentioned in the article, the methodological background is clearly located in the fields of medical ethics and humanities and does therefore not aim to produce a cinematographic analysis.

This paper does not primarily aim to give a close-knit insight into selected movies raising important questions concerning death and dying. It aims to set the stage for taking on a wider angle to reflect on common motifs shared by end-of-life movies to reflect on general tendencies and their implications rather than distinct interpersonal conflicts in the dramaturgy of the movie.

In order to do so, the article will first give an overview of the main criteria for movie selection and the ramifications of the analysis. This will be followed by a contextualization within the scientific framework and a brief literature review. The main analysis will argue that there are common factors that frequently play a role in the character’s decisions at the end of life,^5^ and highlight the centrality of the idea of ‘a life fully lived’ (or the perceived inability to reach this goal). Ultimately, these plotline findings will be discussed in the context of concerns within transdisciplinary health humanities.^6^

## Filmography and movie selection

In order to give an insight into important topics and arguments in the cinematic reception of the end-of-life debate ten of the most representative and impactful movies were selected to serve as example and reference for the main plotline findings. Some of them have already been topic of heated moral discussions, are known to the scientistic community and used as a means of raising awareness to ethical problems in the context of dying or the concept of patient-doctor-relation. In addition to their obvious relevance for the bioethical discourse, many of these movies also share a sort of ‘cult-status’ in society. This idea gets highlighted by the fact that many researchers also put emphasis on the use of these movies for medical training as pointed out by Alexander et al. (2005) and Rosenthal (2018), referring to the term ‘cinemeducation’, that summarizes the increasing implementation of these movies in the context of (bio-)medical education. While there is a broad spectrum of movies that are recurring objects of ethical or philosophical discussions, this paper aims to concentrate on a selection of ten movies. When selecting films for analysis, aside from previous work concerning the related bioethical discourse, another focus was set on international productions or coproductions that reached substantial revenue and/or were nominated for or received renowned prices such as an Oscar or Academy Award to pay tribute to their general popularity. The main criteria for inclusion in the analysis were particularly the occurrence of difficult decisions surrounding death and dying as the key plotline; this ensured that the films analysed went into appropriate depth on the process of dying, on decisions around end-of-life in general, or on assisted dying in particular.

For the purpose of this analysis the term *assisted dying* is used as a broad umbrella concept that encompasses several ethically and legally distinct forms of end-of-life decisions. It includes what is commonly known as *active euthanasia* (ending a person’s life at their request) and *passive euthanasia* (withdrawing or withholding life-sustaining treatment, thereby allowing death to occur) and what is termed *assisted suicide*, as the act of assistance in suicide executed by another person (classified as *physician assisted suicide* if executed by a physician). In addition, this umbrella term also includes further established distinctions that divide the act of euthanasia into *voluntary euthanasia* (per request by a person able to make this decision), *nonvoluntary euthanasia* (concerning a person unable to make this decision) or *involuntary euthanasia* (with objection of the respective person) (see Rubenfeld and Sulmasy 2022). *Assisted dying* as an umbrella term also encompasses the terms *withholding* or *withdrawing life-saving measures*, which focus on the termination of treatment instead of the termination of life. Aside from these categories the analysis also acknowledges the existence of a wide range of alternative or overarching terms with varying emotional and historical charge such as *assistance in dying*,* medical aid in dying*,* mercy killing*, *medical assistance in dying* or *death with dignity*, which highlights the inconsistency in terminology and interpretation of terms along with the complexity of ethical and legal definitions. This broad approach to the definition of *assisted dying* (or *assistance in dying*) concerning end-of-life decisions reflects a key premise of the present analysis: In public perception as well as in cinematic narratives, these various practices are rarely distinguished with conceptual precision. The use of *assisted dying* as an umbrella term thus mirrors how end-of-life decision are commonly portrayed in movies (and therefore perceived by the lay audience). This means, that while such conceptual generalization would be inappropriate and confounding in bioethical or medical contexts, the use of *assisted dying* reflects the fact, that for the general public the distinction of different procedures is less relevant than for the professional observer. Rather than mapping the ethical positions onto any specific legal definitions – which in its terminological complexity will not be in detail be perceived by the lay audience – this analysis therefore aims to explore the underlying narrative patterns, dynamics, and reasoning that shape these portrayals and their potential influence on public perception. In addition, this broad and inclusive approach permits the selection of movies that depict multiple aspects of assisted dying and end-of-life decisions without limitation to a narrowly defined legal category or outcome. This is especially relevant, as the ongoing debate increasingly emphasizes the considerable terminological ambiguity and vagueness in the frameworks concerning federal jurisdictions, bioethics, public health and medical institutions surrounding end-of-life decisions (Jones 2025; Downie et al. 2022; Mroz et al. 2021).

If the selected movies were to be referenced in a simplified representation as any of these terms, the movies *The Farewell Party*, *The Sea Inside*,* Me Before You* and *Silent Heart/Blackbird* would classify as (physician) assisted suicide, while *The Barbarian Invasions*, *Million Dollar Baby* and *Amour* could be labelled active euthanasia.^7^ Passive euthanasia in this list would be represented by the movies *My Life Without Me* and *Whose Life Is It Anyway?* 8 The movie *Still Alice* is a special form for attempted (self-assisted) suicide as the protagonist, who is suffering with early-onset Alzheimer’s disease, uses technology to try and help assist her “future self” in her suicide. Of course, there is a variety of additional movies that (to some extend) meet these criteria or could reasonably be part of this discussion such as *Soylent Green*, *The English Patient*, *The Diving Bell and the Butterfly*,* Wit* or *Igby Goes Down* to name a few famous ones. The selection below, however, was made in order to give a deeper insight into this topic from a larger perspective concentrating on the most relevant movies directly focusing on the protagonist’s end-of-life struggles as the main plotline. Though the depicted storylines contain very different angles on assisted dying, they all present end-of-life decisions such as how the protagonist wants to spend their limited remaining time, whether to seek medical treatment or assisted dying in various forms.

1. **Whose Life is it Anyway?**; USA 1981, J. Badham (comedy, drama)^9^
2. **The Barbarian Invasions** (Les invasions barbares); France/Canada 2003, D. Arcand (satire, comedy, crime, drama, mystery, romance)^10^
3. **My Life Without Me**; Spain/Canada 2003, I. Coixet (with P. Almodóvar) (drama, romance)^11^
4. **The Sea Inside** (Mar adentro); Spain/Italy 2004, A. Amenábar (docudrama, psychological drama, biography, drama)^12^
5. **Million Dollar Baby**; USA 2004, C. Eastwood (boxing, tragedy, drama, sport)^13^
6. **Amour**; France/Austria/Germany 2012, M. Haneke (tragedy, drama)^14^
7. **The Farewell Party** (Mita Tova); Israel/Germany 2014, S. Maymon/T. Granit (comedy, drama, romance)^15^
8. **Still Alice**; USA 2014, R. Glatzer/W. Westmoreland (drama)^16^
9. **Me Before You**; USA 2016, T. Sharrock (feel-good romance, drama, romance)^17^
10. **Silent Heart**; Denmark 2014, B. August (drama)^18^

Remake:^19^**Blackbird**; USA 2019, R. Michell (drama)^20^

The following overview including additional information has been crafted by using the largest international film database, IMDb to provide information about the movies income worldwide as well as the number of nominations and movie prices as a metric of success (Table 1, Internet Movie Database 2025).

**Table 1:** Overview of box office and awards/nominations

Title (Year)	Box office worldwide	Number of prices/nominations	IMDb-Ranking overall (votes)
Whose Life Is It Anyway? (1981)^a^	$8 Million	N/A	7,3/10 (3,7k)
The Barbarian Invasions (2003)	$35 Million	1 Oscar 50 awards 37 nominations total	7,5/10 (31k)
My Life Without Me (2003)^b^	$10 Million	16 awards 15 nominations total	7,4/10 (26,1k)
The Sea Inside (2004)^c^	$44 Million	1 Oscar 69 awards 38 nominations total	8,0/10 (87k)
Million Dollar Baby (2004)	$217 Million	4 Oscars 68 awards 86 nominations total	8,1/10 (743k)
Amour (2012)	$30 Million	1 Oscar 84 awards 111 nominations total	7,9/10 (108k)
The Farewell Party (2014)	$1 Million	9 awards 12 nominations	7,0/10 (2,3k)
Still Alice (2014)^d^	$45 Million	1 Oscar 35 awards 36 nominations total	7,5/10 (147k)
Silent Heart (2014)	$0,5 Million	6 awards 15 nominations total	7,1/10 (2k)
Blackbird (2019)	$2 Million	1 award 1 nomination total	6,6/10 (6,3k)
Me Before You (2016)^e^	$208 Million	6 wins 6 nominations total	7,4/10 (308k)

^a^ The movie is based on the original play by Brian Clark, which has aired as television play as well as turned into a Broadway adaption and has later been published as a novel; it ranked number 4 in the keyword ‘right to die’.

^b^One of the executive producers of *My Life Without Me* is Oscar-winner Pedro Almodóvar, one of the internationally successful icons of Spanish cinema.

^c^Ranked number 1 for the keyword ‘right to die’.

^d^ The movie is based on the 2007 debut novel *Still Alice* by the neuroscientist Lisa Genova.

^e^*Me Before You* is based on Jojo Moyes book from 2012 with the same title; it ranked number 1 for the keyword ‘assisted suicide’ and number 3 for the keyword ‘euthanasia’.

Furthermore, as mentioned in the article:

- I accuse (Ich klage an); Germany 1941, W. Liebeneiner (drama)^21^
- Soylent Green; USA 1973, R. Fleischer (crime, mystery, science-fiction)^22^
- One Flew over the Cuckoo’s Nest; USA 1975, M. Forman (drama)^23^  
- The English Patient; USA 1996, A. Minghella (drama, romance, war)^24^
- Condenado a vivir; Spain 2001, R. Bodegas (drama)^25^
- Wit; USA/UK 2001, M. Nichols (drama)^26^
- Igby Goes Down; USA 2002, B. Steers (comedy, drama)^27^
- Away from her; Canada/UK/USA 2006, S. Polley (drama)^28^
- The Diving Bell and the Butterfly; France/USA 2007, J. Schnabel (biography, drama)^29^
- The Bucket List; USA 2007, R. Reiner (adventure, comedy, drama)^30^
- Iris; USA/UK 2010, R. Eyre (drama, romance, biography)^31^
- Last Cab to Darwin; Australia 2015, J. Sims (adventure, comedy, drama)^32^
- The Room Next Door; Spain/USA/France 2024, P. Almodóvar (psychological drama, drama)^33^

## Theoretical framing and brief literature review

Previous work in film and medical ethics explores a variety of insights on the depiction of ethical dilemmas in movies (Colt et al. 2011; Shapshay 2009; Rosenthal 2018; Bohrmann et al. 2018, Schmidt et al. 2008; Poltrum et al. 2020) often with regards to the potential use for medical education (Alexander et al. 2005; Rosenthal 2020) or the role of old age (Strauß and Philipp 2017). The subject of end-of-life decisions is frequently illustrated by the analysis of one selected movie to explain the conflict of one specific ethical dilemma that may occur in that context, like for example the use of the 2004 production *Million Dollar Baby* to discuss the importance of autonomous decisions (Hawkins 2011), images of disability (Lutfiyya et al. 2009) and the value of human life (Frowe 2009). Moreover, the movie has been used as case study for a model of representation in bioethics (Braswell 2011). Those single-film-discussions exist for a variety of famous movies concerning end-of-life decisions and therefore in their totality offer a close insight to the microenvironment of the interpersonal relations and constellations that lead to certain ethically difficult decisions on screen. Although the abundance of literature on each movie cannot be fully represented here, the mentioned literature above provides an insight into diverse perspectives and approaches of bioethical analysis that exist in reference to these movies.

Previous authors already shed some light on related cinematic aspects of death and dying such as the role of life-threatening illness (Drukarczyk et al. 2014), grief (Schmidt and Roser 2015) and transience of life (Karpf et al. 1993) by analysing the typical patterns and resemblances in the movies instead of creating single-film-analysis. On a related topic Rubenfeld and Sulmasy (2022) offer insights into similarities in the arguments used to promote euthanasia and physician assisted suicide comparing the Nazi propaganda movie *I accuse* (1941) and eight award-winning contemporary feature films including *Million Dollar Baby*,* The Barbarian Invasions*, *The Sea Inside* and *Amour* with a side note to *Me Before You* and *Whose Life Is It Anyway?* This paper aims to extend the spectrum to the cinematic interpretation of end-of-life decisions with emphasis on assisted dying and motifs of the associated storylines on screen.

## Portraying end-of-life decisions – common narratives in the movies and their ethical implication

### The idea of ‘a life fully lived’ – or the perceived inability to reach this goal

*Life for me is over. I cannot do the things I wanna do.*

*I can’t even say the things I wanna say.*

*So, for me … it might as well be over.* 34

According to philosophical discourse human suffering (at the end-of-life) is multifaceted. Beyond mere physical pain, the perceived loss of control over one’s (self-)image through old age and illness or the inability to live according to previous life plans can be profoundly distressing, while being often far more difficult to perceive and articulate (Svenaeus 2020). This dimension of suffering deriving from the idea of a person’s self-narrative and core life values being endangered by being unable to do certain things central to their personal idea of a meaningful life and therefore losing one’s self-identity as well as dignity, can only be understood by a deep understanding of someone’s life as a coherent narrative (Svenaeus 2020). Films offer precisely that: illustrated case studies of personal narratives, offering detailed insights into experiences, emotions, and deep-seated values that can provide a gateway to understanding this existential form of suffering. When regarding death wishes especially in elderly people, there is also a significant risk of medicalization, which may lead to overlooking contextual factors and disregarding their social and cultural embeddedness,^35^ which could be counteracted by adopting a more phenomenological perspective on death wishes (van Wijngaarden et al. 2016). This again can be fostered by using film as a rich source of illuminating material and case studies to support bioethical and philosophical reflection (Schmidt 2000), as it provides deep insight into personal relationships, contextual nuances, and the significance of a coherent life narrative.

One motif that is tangible in almost every movie concerning questions surrounding end-of-life is the idea of ‘a life fully lived’. Regardless of the protagonists age or status, it constitutes a central element that is often referred to in various forms during the plot development. This is due to the idea that a fulfilled life or a not-yet-fulfilled life becomes the central marker as to whether death comes too soon or at an acceptable time. The idea of a fulfilled life is less an ethical concept than that it is implicit in concepts of autonomy and self and deeply rooted in the public debate (Buijsen 2018). In public discourse, however, the idea of a fulfilled life is centrally associated with the issue of assisted dying. The bioethical debate mainly addresses the issue of ‘a fulfilled life’ regarding people with a wish to die that do not suffer from a terminal illness, like it is the case in the recurring discussion in the Netherlands concerning the liberation of assisted suicide for adults at a certain age without terminal illness (Buijsen 2018). However, it is crucial to recognize, that the implications of the concept of a fulfilled life can also create traction with patients that do have a severe prognosis. The concept of a fulfilled life in the movies mainly occurs in two varying manifestations. For one there is the stereotype of the aged, highly educated, and well-situated elderly that near his/her last days reflects on the ups and downs of his/her life, making it clear that he/she has lived a full life both in terms of satisfying personal relations and/or by pursuing his/her dream career. Through stories and memories told by loved ones, throwbacks or sometimes an intertwined storyline with parts of the old and young protagonist (as particularly prominent in the movie *Iris*) the viewers get to reflect on the protagonist’s life as fulfilling and contempt. Therefore, the desire for or acceptance of death aligns in the spectator’s view as an extension of the willpower and autonomy shown by the protagonist in the past in his/her quest to lead a self-directed life.

A different angle in which the idea of ‘a live fully lived’ is used as a driver of the dramaturgic conflict in end-of-life decisions in the movies is through showing in a similar way the protagonist’s past life up to a point where an accident or illness inhibits the protagonist to live up to his or her idea of what a meaningful life should look like. By creating this cut in the characters biography, that leaves them with uncertainty and a sense of inability to achieve former goals like being successful in the pursuit of their career or living an autonomous life (assisted) dying increasingly becomes the apparent only option to regain a sense of autonomy and agency due to external factors. Rubenfeld and Sulmasy (2022) describe this specific portrayal of storyline development as “the depiction of lost talent as the loss of a claim to life”.

In the movie *Amour* this feeling gets expressed by the around 80-year-old Anne, who recently suffered a stroke, when she explains to her husband who caught her trying to commit suicide at the open window: *I just see no reason to keep on living. I know I will only get worse. Why should I do this to us? To you and me.*^36^

Especially young protagonists frequently exhibit a perspective that implies ‘life has nothing left to offer’. However, this view is not a reflection of contentment but rather a sense that life with this particular condition or situation does not align with their vision of a fulfilling existence. The idea, that the challenges inherent to the new circumstances hinder the ability to achieve any goals that are personally associated with ‘a well-lived life’ is apparent in the movie *Million Dollar Baby* when the former rising boxing star tells her trainer: *I can’t be like this Frankie. Not after what I’ve done. I’ve seen the world. People chanted my name.*^37^

Similarly in *The Sea Inside* the protagonist explains: *If I accept the wheelchair*,* I accept the remnants of my former freedom.*^38^ Ramón (a character inspired by the life of Spanish writer Ramón Sampedro) who is rendered tetraplegic due to a diving accident in his youth expresses his desire to legally end his life and regain a sense of control over his own life via a 30 year-long legal fight.^39^ Ramón’s main argument contains, that as he feels that he is incapable of truly living in the way he desires, he therefore demands the right to die on his own terms.

A particularly emotional moment of anti-climax can be found in *Me Before You* when Will Traynor, who has been rendered tetraplegic due to an accident, and his employed personal assistant Louisa (Lou) Clark kiss on a romantic beach-vacation, leading the audience to expect a classical happy ending. Lou, who has arranged the holiday in order to convince Will to abandon his secret plans to end his life believes that her love and company have ‘cured’ Will of his wish to die. Just when the developing love story starts to unfold, he rejects her with the words *I get that this could be a good life. But it’s not my life. It’s not even close. You never saw me before. I loved my life. I really loved it. I can’t be the kind of man who just accepts this*.^40^ In the following he not only hints to his former perfect life as a young, famous and rich businessman, but also implies, that he is afraid that Lou would regret spending her life with him or pity him due to his disability, creating a very difficult atmosphere concerning the portrayal of disability, especially as the general conception of the storyline works to encourage the audience to accept Will’s decision for euthanasia as an autonomous and almost logic act of freedom (Botha and Harvey 2022).^41^

Often the emphasize of young protagonists’ moral struggles concern the inability to create their future life according to their ideals, while the focus for older protagonists rather relates to the plotline development featuring hints of the idea of already having had a full life. Aged protagonists therefore are often portrayed in a more content way, showing that life has nothing new left to offer them that they have not experienced before. This sentiment becomes apparent in the movie *The Farewell Party*. The 72-year-old Yehetzkel lives with his friends and his wife Levana, who has been diagnosed with dementia, in a supportive housing facility and constructs a machine to allow a friend suffering from terminal stages of cancer, to end his life by pressing a button. Already during the funeral, outsiders begin to press him regarding the use of his euthanasia apparatus, leading to moral discussions within the involved friend group about the act of assisted suicide. At the end of the movie, Yehetzkel first prevents his wife’s attempted suicide ultimately to then enable her to have an assisted suicide using the self-made apparatus. As the title implies the overall sentiment is the celebration of dying as an autonomous act and a last highlight after a life filled with love and kindness.

Yet this difference between the age groups is not strict and sometimes both perspectives coexist. In mid-aged protagonists, who had been healthy up to this moment and share many precious memories from their former life while being torn between the notion of having had a beautiful life and being afraid of the perceived inability to do so in the future, this juxtaposition is especially tangible. An example for this concept is Rémy, the protagonist of *The Barbarian Invasions* who reflects upon his formerly illustrious life as well as unfulfilled dreams in the company of family and his old friends after being diagnosed with terminal cancer, before ultimately with the aid of his loved ones dies through a heroin overdose. 

It is very curious, that most end-of-life movies feature a certain kind of environment that mainly focuses on highly educated academics (Rubenfeld and Sulmasy 2022), often in a teaching position and with beautiful scenery like beach houses or city lofts. This constitutes a similar tendency than described by Holzhauser and Frewer (2017) regarding movies featuring dementia and is not representative of standard living circumstances of people facing severe illness or devastating prognosis. Under the ten listed movies there are only two exceptions with *My Life Without Me* featuring an uneducated young cleaner and mother/housewife suffering from ovarian cancer who is living in a trailer park and the first half of *Million Dollar Baby* featuring a rising-from-poverty young boxing-star. The general depiction of wealth and power emphasizes the former ‘perfection’ and high functional level of the protagonist’s life that gets destroyed by illness or frailty and is used as highlighter and legitimization of the protagonists wish to die.

For characters of all age groups there are some further similarities. When confronted with death (unavoidable through pending illness or self-chosen) the main characters often start creating a mental or written Bucket list with moments they desire to (re)experience; there even is a 2007 dramedy movie called *The Bucket List* featuring exactly this phenomenon by following the last days of two terminally ill patients. At the same time there is often room for sorrow about the past concerning personal relations or other regrets about not being able to leave a mark to the world or having found sense in one’s life. Other similarities are the need to care for the people they leave behind in a variety of ways, the most extreme one being the search for a ‘replacement’ for their partners as this is the case in the movies *Silent Heart/Blackbird* and *My Life Without Me.*^42^

### Social isolation and negative experiences within healthcare as indicators of the inability to live a meaningful life

Yehetzkel: *I would never in my life leave you in a place like this.*

Levana: *Don’t you remember what she said? I won’t even*.

*be aware of where I am*,* so what does it matter?*

Yehetzkel: *It matters to me. We would be in there together.*

Levana: *But I would still be alone.*^43^

A recurring motif in end-of-life decisions at the movies is the circle of isolation and the desire to die. This often begins with the character mentioning thoughts concerning death and assisted dying to their loved ones or medical staff. Instead of embracing these shared thoughts as an opportunity to open the conversation about fears, wishes and current struggles it frequently is portrayed as a starting point of accusation towards the protagonist as it is perceived as ungrateful from the private or professional caretakers’ viewpoint. Therefore, the desire to die in itself if it remains unaddressed becomes the isolating force thus reinforcing itself. The recurrent rejection of a wish to die is particularly prominent in *The Sea Inside*, when the tetraplegic Ramón repeatedly must explain to members of his family, his lawyer, a clerk, the court and even strangers why he wants to end his life instead of trying different devices to help compensate his paralysis. While the first reaction to his desire is frequently rejection, the continuous conversation itself during the plot development helps to dissolve conflicts e.g. within the family, even if his decision is still not approved by everyone.

*Now without self-pity*,* I am no longer someone to love. I am an object that has to be taken care of for the rest of his life.*^44^ This dark self-image gets drawn by Ken Harrison, a sculptor that has been paralyzed from the neck downwards after a car accident, as he fights in *Whose Life Is It Anyway?* for his right to die and recurrently refers to himself and patients in similar condition as ‘vegetables’. This stresses the unsettling potential in films’ depiction of people impaired by old age, illness or in this case disability struggling with a subjective sense of losing relevance or ‘value’ to their families or to society at large, due to the possibility that audiences may consciously or subconsciously associate these characters’ desire to die with the feelings they themselves may experience in this regard (Wagensonner 2023). A frequent concomitant of this motif around protagonists’ sense of irrelevance as humans is their loss of legal control over their own affairs or a more subtle disempowerment that takes place verbally or at the hands of wider society. This feeling of irrelevance in the movies shows the potential to serve as a sort of catalyst towards even greater isolation from loved ones and their former life thus reinforcing the desire to die. As the wish to die or even the thought about death does not get validated and honoured by friends and family and often even gets tabooed this reinforces the feeling of isolation and loss of significance.

Moreover, not only the anxiety surrounding difficult conversations about death wishes or the wish for treatment limitations, but also the fear of stigmatisation concerning the underlying condition can create a feeling of seclusion. When it comes to neurodegenerative conditions such as in the case of *Still Alice* a form of early onset Alzheimer’s, there is often a significant concern of social ostracism that can reinforce social isolation. This becomes apparent when Alice refuses dinner invitations out of fear of destroying the illusion of her academically successful, eloquent self. In a fight with her husband, she expresses her feelings as follows:

Alice: *I wish I had cancer.* Husband: *Don’t say that.* Alice: *No I do*,* I mean it. I wouldn’t feel so ashamed. When people have cancer*,* they wear pink ribbons for you […] and you don’t have to feel like some sort of a social … I can’t remember the word.*^45^

Aside from the movies potential to help identify stigmata, illustrations of isolation due to disability or illness can through empathic identification help raise awareness for these societal problems.

In other variations of this motif of social isolation the secret of planning one’s suicide because of pending loss of cognitive functions as in *Still Alice* or hiding one’s terminal illness from family and friends as in *My Life Without Me* forces an invisible barrier between the protagonists and their clueless families. The protagonist Ann in *My Life Without me* describes the resulting feeling in a self-dialogue as following: *Alone. You’re alone. You’ve never been so alone in your life. Lies are your only company.*^46^

An often-overlooked aspect that is seldom reflected upon in movies concerning end-of-life decisions is the potential psychological value and significance of the wish to die as a coping mechanism to alleviate anxiety and reduce stress. A wish for death is a multifaceted phenomenon that can serve as both an expression of not wanting to endure the current quality of life (or perceived lack of it) but also as a potential way out from the anticipated overwhelming pain of the mortal existence. Ohnsorge et al. (2014) for example differentiate between functions, reasons and meanings leading to a complex connection to motivation and intention concerning a wish to die and characterize no less than nine different meanings of a wish to die, including “to end a situation that is seen as an unreasonable demand” and “to spare others from the burden of oneself”. Common reasons according to this review are “appeal”, “vehicle to speak about dying” and “re-establishing agency” as well as “manipulation”. This insight, when applied to the ethical dimension of frequent representation of wishes to die, stress that the binary division in morally right or wrong decisions on screen fail to captivate the complexity of the conversation about wishes to die. It is also important to consider, that a desire to die can also arise in the context of (treatable) conditions such as depression or other variations of active or passive suicidal thought, which might sometimes be difficult to distinguish. As this important aspect is rarely explored in the depicted movies it is therefore rarely brought into the cinematic conversation. The various manifestations of (social) isolation as depicted above are often depicted as one particular obstacle in the characters pursuit of ‘a life fully lived’ as being perceived as a valuable part of a community (in family systems or in terms of workplace or friendship groups) is portrayed as a deep routed human need that ceases to be fulfilled.

Another apparent hindrance of experiencing a well-lived life that is often reflected upon in the cinematic narrations are adverse experiences within the healthcare systems especially concerning institutions such as hospitals or nursing homes or their representatives. The euphemism of ‘going home’ is sometimes used to refer to dying; the notion of an ‘eternal home’ after death is a comforting image facing the loss of a loved one (Auchter 2017). A powerful sense of contrast emerges between these ideas and the general cinematic representations of care homes, hospitals, or residences for older people in the analysed feature films. In the movies this represents as either the protagonist’s general scepticism towards hospitals and care homes or the stylisation of health-caretakers such as nurses and especially doctors as ‘the enemy’ who contracept the patient’s idea of living a fulfilling life or based on the perceived inability to do so experiencing a ‘dignified’ death. Aside from the prevalent negative connotation of characters coded as medical professionals in many movies, the counterintuitive lack of physician displays in other movies concerning (physician) assisted dying or euthanasia should also be noted and might even hint to changes in the public perception of these matters as no longer primarily medical responsibilities (Rubenfeld and Sulmasy 2022).^47^

*I’m afraid there is nothing we can do*^48^ stammers the doctor in *My Life Without Me* while explaining to the 23-year-old Ann, that she has end-stage ovarian-cancer, all while avoiding eye-contact and at the same time delivering an almost impossible to forecast prognosis: *Two month… maybe three*^49^. In less than a minute the physician therefore vividly illustrates a textbook version of ‘how *not* to deliver a devastating prognosis’.^50^

The dramatic announcement of the remaining lifetime as a key scene (and as a foundation of decision-making concerning end-of-life choices) is an especially delicate issue, as for one, as a scoping review by Ferrand et al. (2022) shows, physician prognostication tends to be rather pessimistic and second the inaccuracy and interindividual variability of prognostication is not evident to the general public.

*Promise me one thing*,* okay? […] That you’ll never take me to the hospital again*^51^ is the strong request stroke-survivor Anne asks of her husband George in the movie *Amour* after her first hospital stay. In later scenes an increasingly desperate George even threatens Anne with hospitalization and parenteral nutrition as she refuses to drink. His despair mountains in his forcing Anne to drink and even hitting her in the face as she spits.

There is an overall obvious negative depiction of hospitals and doctors as a cold and unhelpful system often except for one or two female nurses or sometimes (female) doctors that take heart to listen and tend to the person rather than the patient (concerning the evolving presentation of nurses see Wulff 2023).

This overall negative display comes with an apparent non-existence of palliative medicine in the movies. According to the WHO definition of palliative care (WHO 2002) palliative medicine can be described as “an approach that improves the quality of life of patients and their families facing the problems associated with life-threatening illness, through the prevention and relief of suffering by means of early identification and impeccable assessment and treatment of pain and other problems, physical, psychosocial and spiritual” and can almost be seen as a description of everything the healthcare workers in most of the movies are not showing. In this way a relevant difference between the film’s portrayal of medical care and today’s palliative medicine approaches becomes clear.

Moreover, movie-plotlines emphasize the need for a feeling of control. This feeling of fading agency in the movies is often not only caused by the life-threatening or life-changing illness or ailment itself but by the hospital and its representatives. In other words, the fear of dying from cancer or dying itself is often portrayed as less important than the fear of dying ‘here’ (in the hospital/care home) or dying while being dependent on machines and tubes as the following quote illustrates: *In a few weeks it’s possible she won’t be able to move at all*,* to talk or even to swallow. […] She’d be surrounded by machines to help her breathe and to remove the saliva and so on. And we’d have to feed her through a tube. […] She doesn’t want that. And she has decided that now is the time to do it*,* while she is still in charge of what happens to her*^52^ explains the grandfather in *Blackbird* to his teenage grandchild regarding the upcoming assisted suicide of his wife who is suffering from ALS (amyotrophic lateral sclerosis).

The often simplistic wording and presentation in the movies highlight the need for public awareness, as well as for understandable and accessible information about the realities of palliative medicine and of practices like the use of ventilators and feeding tubes.

### Age and time as a source of grief and as a main driver of the dramatic climax

*Rémy: It sounds paradoxical, but the older you get, the more you cherish life. […]*

*No one knows what will happen to them in the future. Except me now. I know it.*

*Nathalie: Are you afraid of it?*

*Rémy: Oh yes*,* very much so. I don’t want to stop living.*

*You can’t imagine how much I loved life*.^53^

*I didn’t realize I had so little time. Actually*, *time is the*

*one thing I haven’t had enough recently.* 54

Central aspects of the dramaturgy underlying films in the area of analysis include the matter of the time an individual with a life-threatening illness such as cancer still has to live, and of the passage of time that remains to a protagonist to make an autonomous decision on suicide or assisted dying before, as in *Still Alice*, *The Farewell Party* or *Silent Heart/Blackbird* a neurodegenerative condition renders autonomous action impossible. But how much time is enough time in this context? This question, sitting at the heart of the conflict depicted in these films, is of multi-faceted currency in the societal debate around assisted dying. This holds especially considering the rising weight accorded to patients’ autonomy and in view of demographic change. Ambiguity and complexity are inherent to the question itself, which carries the dual implication of ‘How much time is enough time in a life?’ and ‘How much time does it take to arrive at a decision for or against assisted dying?’. One burning question-within-a-question in this regard is that of whether the inherent irrevocability of this decision means there is ever ‘enough’ time for its making. Public discourse on this matter places substantial emphasis on the autonomy of terminally ill people or people with severe disabilities, especially those of younger age. Reflecting this focality of concern, more recent films on assisted dying have increasingly tended to feature younger patients (Schmidt 2017), with a concomitant inclination to aestheticize death (ibid.), in line with a societal adulation of high achievement, success and youth that effectively constitutes a subtle form of ageism (see, in particular, Frewer et al. 2020). Both in public debate and in films, discussions of issues around assisted dying frequently relate to cancer, neurodegenerative conditions or serious accidents that leave an individual permanently seriously disabled with conditions such as tetraplegia (Wulff 2016). The selected movies include an age span from a former healthy 23-year-old mother of two suffering from ovarian cancer to an estimated 80-year-old wife suffering from a stroke. While there are many movies focusing on the specific dramaturgy concerning old age (Wagensonner 2023; for general portrayals of the elderly see also Markov and Yoon 2021) it is apparent, that especially famous movies focus on younger protagonists facing end-of-life decisions as described above. From a different angle this raises the question why movies around end-of-life decisions concerning young protagonists seem to be especially successful. Beside the aesthetic implementations, the presentation of a young protagonist also has the potency of creating more dramatic effect due to different living circumstances and factors like higher probability of technological advancement and cure in the future. While older protagonists mainly struggle to keep their agency and be respected as still capable of making their own decisions (Wagensonner 2023), the main struggle for young protagonists often is to confirm the finality of their decisions, as there seems to be an even greater need to ‘proof’ that in the many years they might have left to live they would not change their inherently irrevocable choice.

In *Silent Heart/Blackbird* the overpowering role of the remaining time is visually forced upon the viewer by a variety of clocks, watches, and timepieces in the family’s beach house, while the family prepares the assisted suicide of the mother Anne, who is suffering from the neurodegenerative condition ALS (amyotrophic lateral sclerosis). In consultation with her husband (who is a general practician) she decides to die while it is still possible to make her death appear as suicide before any severe impairments are present. While the whole extended family over and over discusses the question of whether the ‘right time’ has yet arrived, many scenes include multiple ticking timepieces at once enhancing the pressing nature of this decision.

*I am not suffering. I am struggling.*

*Struggling to be a part of things*, *to stay connected to who I once was.*

*So*,* live in the moment I tell myself.*

*It’s really all I can do, live in the moment.* 55

The unique significance of the present moment is accentuated by the general nature of films, as they allow viewers to experience and connect with the immediate experiences of the characters. The past on the other hand is only accessible through memories; the future, as seen in the moment of watching the movie, still lies in the audience’s own future. Therefore, the conflict of time extends beyond the cinematic narrative level. A typical feature films average running time of approximately 90–120 min is the time available to the audience for attaining insight into the protagonist’s life and emotional landscape to such an extent as to enable it to reconstruct the internal processes that lead someone for instance to undergo assisted dying (Wagensonner 2023). This is reinforced by the fact that a typical feature film represents a starkly limited space for the exploration of end-of-life decisions, if we hope it will offer answers to the questions they raise.

## Through the lens of reality: conclusions speaking to the concern of transdisciplinary health humanities

There are several insights that we can extract from the main common threads as caretakers and healthcare professionals about fears, problematic tendencies and pending issues that are reflected upon in the movies. A concern that the totality of movies appoints to is the potential intersection of the idea(l) of ‘a life fully lived’ and society’s attitude towards the elderly (in terms of ageism) but also towards severely impaired persons. This might lead to the narrative of age and disability as legitimate argument for/against assisted dying in the public discourse considering society’s pursuit of perfection and aesthetics as portrayed by (social) media as well as many of the movies. At the same time depiction and representation of these conditions and the danger of isolation if illustrated and reflected upon in a sensible way might help raise greater awareness towards these topics. Moreover, it is important to reflect upon the fact that movies create a constant need to challenge the way we think about death and dying related to different age groups. While there naturally might be differences between specific concerns due to varying circumstances and living arrangement etc. there is no one type fits all set of problems and solutions to death-wishes in different ages.

The overall message included in many movies is an appeal to the general need for a socially and emotionally supporting environment and the human touch at the end of life. As explored in this article, the storylines on screen seem to frequently highlight the need for improvement of hospital culture as well as nursing homes. These institutions need to be ‘real homes’ to their residents or patients, providing a sense of comfort and dignity can be read as the underlying subtext. On the one hand this exaggerated and simplistic stylisation of the healthcare system as the enemy that prevents individuals from experiencing a dignified and self-directed death as natural conclusion after an autonomous and well-lived life simply provides an interesting and provoking storyline. One might even assume, that the producers’moral convictions concerning assisted dying are less relevant to the characters (and movies) ending than the need for a thrilling and dramatic effect that speaks to all human emotions. After all a heroic fight against the faceless institutions and cold-blooded doctors might create more tension (and therefore income) than the depiction of well-executed palliative medicine and well cared-for dying patients. On the other hand, it can still be interpreted as a symptom of a lack of knowledge and the multitude of fear and uncertainty surrounding the issues of palliative medicine as well as various forms of assisted dying. While the cinematic exclusion of palliative medicine therefore might be primarily due to the purpose of entertainment and creating dramatic effect, it may still be regarded as representative for the lack of comprehensive and sufficient ambulatory or inpatient palliative care in many countries.^56^

In order to further explore the potential intentions behind the negative narratives around institutions and the healthcare system more research into this specific field is needed. Especially when it comes to conclusions concerning hospital and nursing culture a comprehensive comparison between the depicted circumstances and the reality of daily life in institutions might be an important next step.

An interesting aspect that is explored by Rubenfeld and Sulmasy (2022) is the idea of an absence of physicians’ portrayal in movies concerning euthanasia as indicator for a societal change in the perception of medical authority over the moral discourse shifting to a point of view where medical ethics is dictated by society and state and merely executed by the medical profession. This also represents an important starting point for further research into the area of how the presence and role of medical professions in movies concerning assisted dying might have changed over the years and whether or how this relates to reality.

In addition to the need for improvement of hospital and nursing culture and the combat of ageism, the analysis also identifies the fear of social isolation as a key factor impending individuals from achieving ‘a life fully lived’ and therefore influencing end-of-life decisions. This underscores the importance of greater awareness regarding the emotional and social embeddedness of individuals. A person’s sense of meaningful contribution to the family or community and being perceived as a valuable member of society plays a crucial role in end-of-life decisions. Moreover, the experience of social isolation at the end of life can inhibit the opportunity for meaningful conversations about one’s personal idea of a fulfilled life.

However, a general conclusion that might be drawn from this issue is, that by opening the conversation to difficult decisions about advanced directives, ‘allowing death’ or refusing life prolonging measures, it might prevent in some cases the wish for extreme measures such as (assisted) suicide. As the dualistic view that is being portrayed in movies of being tied to tubes in the hospital or regaining agency in taking one’s life gets dissolved and the option of nuances and middle ground gets visible, opening conversation to different options might even increase autonomy instead of reducing it. In addition, the movie itself as an object creates a starting point for discussion within families, friend groups or professionals and therefore can encourage open discussion about difficult decisions at the end of life as well as personal values and beliefs. It is crucial to encourage open conversation and confrontation about dying and death to help individuals express their concerns and needs without judgment, to prevent the spiral of sense of guilt and isolation from creating an insurmountable barrier. Amidst the plethora of ambiguous and confusing legislation and regulations, we must ensure that families and healthcare workers can openly discuss end-of-life wishes without fear.

While the ten selected movies already give a first comprehensive perspective on aspects of end-of-life decisions and cover a broad spectrum of different ways of assisted dying in protagonists of all ages and with a variety of underlying issues, the different aspects within the article highlight the need for further research in many topics that build upon and extend the ideas presented in this article, delving into the ambiguity of the portrayal of institutionalization, problematic tendencies in healthcare and ageism as reflected upon in movies.

The nearly four-decade range of movies examined in this article (1981–2019) reflects not only the enduring cinematic interest in emotional challenges surrounding end-of-life discussions, but also illustrates that these decisions remain a topic of cultural engagement and public concern. This underscores the ongoing need for open dialogue and ethical reflection on end-of-life decisions. This becomes even more apparent when considering the message of the 1973 cult classic *Soylent Green*, one of the defining productions of early science fiction and among the first eco-dystopias. Like *The Room Next Door*, it is set in New York (in the year 2022) and serves as a warning metaphor for the consequences of global warming, overpopulation and resource scarcity. These (very current) problems have not only led to the legalization and institutionalization of euthanasia but also to the disturbing practice of turning human corpses into the nutrition supplement known as ‘Soylent Green’. Though stylistically different, more than 50 years after the release of *Soylent Green*, *The Room Next Door* continues to echo similar anxieties about unresolved societal and environmental issues, reinforcing the special capacity of stories in film to “stimulate and enhance philosophical and bioethical reflection” and to serve as “theoretical laboratory” (Roduit et al. 2018). The fact that such movies continue to be produced and debated in the way they are indicates that society grapples with these complex issues. It serves as a reminder that there is much work to be done, as the big screen persists in its mission to challenge (and influence) the public discourse.

## Data Availability

Not applicable.

## Footnotes

1
*The Room Next Door*, 01:24:15.

2 Pedro Almodóvar was also involved in the 2003 production *My Life Without Me*, featuring a 23-year-old mother’s quest for a meaningful life after being diagnosed with end-stage ovarian cancer.

3 The movies general potential to spin these developments further and therefore serve as socio-cultural experimentation field for an alternative dying culture is particularly apparent in the genre science-fiction (Wagensonner and Frewer 2021).

4 A helpful introductory insight into the vast body of work on empathetic processes during film reception is provided by the working bibliography of Wulff (2003).

5 While not all motifs are found in every single movie concerning end-of-life decisions and assisted dying, they are often found coexisting and thus self-reinforcing.

6 The first author has previously explored related aspects of this topic with the focus on autonomy of the elderly in German feature films in Zeit zu gehen? Schlaglichter zur Autonomie Älterer in deutschsprachigen Spielfilmen zur Sterbehilfe, published in “Best Aging?” Arbeiten für ein gutes Alter(n) aus menschenrechtlicher und moralischer Sicht (2023), 77-120.

7 While the first three movies could qualify as cases of voluntary euthanasia, *Amour* could arguably be labeled nonvoluntary euthanasia.

8 It is necessary to distinguish, that the decision to withhold treatment in *My Life Without Me* is rather due to the unavailability of a specific, effective treatment for the protagonist’s end-stage ovarian cancer than to a conscious refusal of said treatment, whereas in *Whose Life is it Anyway?* the concerning action is the withdrawal of previous life-sustaining treatment such as ventilation. This is a good example for the shortcomings of certain terminology as it constitutes fundamentally different circumstances.

9
https://www.imdb.com/title/tt0083326/?ref_=nv_sr_srsg_0_tt_8_nm_0_q_whose%2520life.

10
https://www.imdb.com/title/tt0338135/?ref_=fn_al_tt_1.

11
https://www.imdb.com/title/tt0314412/?ref_=fn_al_tt_1.

12
https://www.imdb.com/title/tt0369702/?ref_=fn_al_tt_1.

13
https://www.imdb.com/title/tt0405159/?ref_=nv_sr_srsg_0_tt_8_nm_0_q_million%2520dollar%2520bab.

14
https://www.imdb.com/title/tt1602620/?ref_=fn_al_tt_1.

15
https://www.imdb.com/title/tt3163304/?ref_=nv_sr_srsg_0_tt_7_nm_1_q_the%2520farewell%2520party%25%2B.

16
https://www.imdb.com/title/tt3316960/?ref_=fn_al_tt_1.

17
https://www.imdb.com/title/tt2674426/?ref_=fn_al_tt_1.

18
https://www.imdb.com/title/tt3335048/?ref_=nv_sr_srsg_0_tt_7_nm_1_q_silent%2520heart.

19
*Blackbird* is the 2019 remake of the Danish movie *Silent Heart*. Therefore, they are in the following mentioned together, as though there are minor differences, the main plotline and events are identical.

20
https://www.imdb.com/title/tt8717446/?ref_=fn_al_tt_1.

21
https://www.imdb.com/title/tt0033750/?ref_=fn_al_tt_1.

22
https://www.imdb.com/title/tt0070723/?ref_=fn_al_tt_1.

23
https://www.imdb.com/title/tt0073486/?ref_=nv_sr_srsg_0_tt_8_nm_0_q_one%2520fle.

24
https://www.imdb.com/title/tt0116209/?ref_=nv_sr_srsg_3_tt_6_nm_2_q_the%2520englis.

25
https://www.imdb.com/title/tt0293046/?ref_=fn_al_tt_1.

26
https://www.imdb.com/title/tt0243664/?ref_=nv_sr_srsg_1_tt_4_nm_3_q_Wit.

27
https://www.imdb.com/title/tt0280760/?ref_=fn_al_tt_1.

28
https://www.imdb.com/title/tt0491747/?ref_=fn_al_tt_1.

29
https://www.imdb.com/title/tt0401383/?ref_=fn_al_tt_1.

30
https://www.imdb.com/title/tt0825232/?ref_=fn_al_tt_2.

31
https://www.imdb.com/title/tt0280778/?ref_=fn_al_tt_1.

32
https://www.imdb.com/title/tt3680410/?ref_=fn_al_tt_1.

33
https://www.imdb.com/title/tt29439114/?ref_=fn_all_ttl_1.

34
*Whose Life Is It Anyway?*, 01:19:45. The paralyzed sculptor Ken in a heated conversation to the young doctor who passionately tries to reignite his will to live.

35 Research in this area highlights the significance of these contextual factors. Quality of life for example has been found to be the main determinant for a wish to die in elderly patients, as a study conducted in an internal medicine ward has shown (Bornet et al. 2020).

36
*Amour*, 00:42:59, *translated from the German version.*

37
*Million Dollar Baby*, 01:47:56. The rising boxing star Maggie after asking her former trainer for active euthanasia.

38
*The Sea Inside*, 00:07:43, *translated from the German version.*

39
*The Sea Inside* is the second film adaption of Ramón Sampedro’s life, succeeding the 2001 drama *Condenado a vivir.*

40 *Me Before You*, 01:28:46.

41 While the movie does not show the actual death scene, a letter from Dignitas (a legal Swiss organization), which can be seen in previous shots, indicates, that Will might receive assistance to end his life in the form of assisted suicide. As he is tetraplegic but still able to swallow on his own, it is a possible option. Nevertheless in scholarly work the ending is often referred to as euthanasia, as the precise technical circumstances are not specified and remain unclear, especially to the average untrained spectator.

42 This motif can also be found in related movies like the Canadian dementia movie *Away from her*, which has been nominated for two Oscars.

43
*The Farewell Party*, 00:04:13. Levana, who has been diagnosed with Dementia argues with her husband Yehetzkel about the potential transfer from their supporting housing to the nursing home after visiting a senior care facility.

44
*Whose Life Is It Anyway?*, 00:34:09. Ken Harrison to his girlfriend as he is breaking up with her because he fears that she is only staying with him out of a sense of obligation and empathy.

45
*Still Alice*, 00:35:04.

46
*My Life Without Me*, 00:28:50.

47 Interestingly this finding also correlates with a recent increase in more negative or ambiguous displays of physicians especially in the related field of medical dramas, slowly replacing the former omnipresent depiction of the stereotypical goodhearted omniscient clinician (Cambra-Badii et al. 2020).

48
*My Life Without Me*, 00:14:44.

49
*My Life Without Me*, 00:15:09.

50 There are, however, few more positive movie-examples of physician representation, like the transformation of the socially uncomfortable doctor in *My life without me* who later agrees to take care of birthday messages that his patient has created for her minor daughters in exchange for her agreeing to regular check-ups so he can register pain medication.

51
*Amour*, 00:25:33, translated from the German version.

52
*Blackbird*, 00:22:14.

53
*The Barbarian Invasions*, 00:52:00. Excerpt from a conversation between the protagonist Rémy and the junkie Nathalie who gets paid to provide him with heroin as a means of pain control.

54
*My Life Without Me*, 01:36:14. The young protagonist Ann in an audiotape that she made for her affair Lee to explain her death.

55
*Still Alice*, 01:07:20. The protagonist Alice in a patients-perspective-speech at an Alzheimer’s association conference.

56 A study by Clark et al. (2020) suggests that as of 2017 only 14% of the population worldwide had access to the highest level of palliative care provision, highlighting the relevance of this void.
